# Management strategies of pediatric ipsilateral olecranon with associated radial neck fractures based on multicenter experience

**DOI:** 10.1186/s13018-021-02373-x

**Published:** 2021-03-30

**Authors:** Jin Li, Sheng Ping Tang, Guo Xin Nan, Ming Li, Shun You Chen, Hai Bo Mei, Jing Fan Shao, Fei Jiang, Rushyuan J. Lee, Xin Tang, Chinese Multicenter Pediatric Orthopedic Study Group (CMPOS), Jin Li, Sheng Ping Tang, Guo Xin Nan, Ming Li, Shun You Chen, Hai Bo Mei, Xin Tang

**Affiliations:** 1 Department of Orthopaedic Surgery, Union Hospital, Tongji Medical College, Huazhong University of Science and Technology, Wuhan, 430022 China; 2 Department of Pediatric Orthopaedic Surgery, Shenzhen Children’s Hospital, Shenzhen, 518046 China; 3 Orthopedic Center of Children’s Hospital of Chongqing Medical University, Chongqing, 400014 China; 4 Department of Orthopeadics, Fuzhou Second Hospital, Xiamen University, Fuzhou, 350007 China; 5 Department of Orthopeadics, Hunan Children’s Hospital, Changsha, 410007 China; 6 Department of Pediatric Surgery, TongJi Hospital, TongJi Medical College, Huazhong University of Science and Technology, Wuhan, 430030 China; 7 Department of Pediatric Surgery, Dalian Medical University affiliated Dalian Children’s Hospital, Dalian, 116012 China; 8 Department of Orthopaedic Surgery, Bloomberg Children’s Hospital, Johns Hopkins Hospital, Baltimore, Maryland USA

**Keywords:** Ipsilateral, Olecranon fracture, Radial neck fracture, Monteggia equivalent lesions, Classification, Children

## Abstract

**Background:**

The ipsilateral olecranon with associated radial neck fractures does not include in the Bado classification of Monteggia fractures and equivalent lesions. The primary aims of this retrospective multicenter study were to characterize this type of injury and, noting its unique properties, evaluate the results of the treatment, determine the prognostic factors that influence the radiological and clinical outcome, and also give treatment strategies.

**Methods:**

Between July 2011 and July 2016, forearm fracture patient charts were retrospectively reviewed from seven pediatric trauma centers. Patients diagnosed with ipsilateral olecranon with associated radial neck fractures and followed up for at least 24 months were included. Fracture characteristics, treatment, outcome, and complications were assessed. The clinical outcome of treatments was evaluated by the Mayo Elbow Performance Score (MEPS) and the Flynn criteria. Fisher’s exact test and ANOVA test were used; significance was defined as *P* < 0.05.

**Results:**

One hundred thirty-seven consecutive patients (54 girls and 83 boys) from 8292 forearm fractures patients, the mean age of 7.5 years (1.5 to 14.8), with fractures of the ipsilateral olecranon with associated radial neck fractures were identified. One hundred twenty-five patients had radiologic and clinical follow-up. According to a simplified classification system with “operate” and “don’t operate” groups, including five subtypes proposed in this study, ipsilateral olecranon with associated radial neck fractures subtypes could be classified with significantly different characteristics and outcome in treatment and complications.

**Conclusions:**

Fractures of the ipsilateral olecranon associated with the radial neck are not so rare as previously reported. Complications and poor outcomes were easy to encounter without knowing this type of fracture. Appropriate treatment strategies could be made according to a simple classification system based on the treatment result of follow-up.

**Level of evidence:**

Retrospective comparative study; Level III

## Introduction

Bado classification for Monteggia fracture did not include the pediatric ipsilateral olecranon or shaft of ulna fracture with associated radial neck fracture with or without dislocation of the radial head [1–5]. Though it is a rare injury, such Monteggia equivalent injury is reported in the previous literature [6–10] and commonly described as a greenstick fracture of the proximal ulnar metaphysis, associated with radial neck fracture [10–13]. Because of the rarity of this kind of injury, there was no classification to characterize the subtypes, or treatment strategies recommended. The treatment results of such kind of injury were usually poor [10, 11, 13].

The primary aim of this retrospective multicenter study was to characterize this type of injury and, noting its unique properties, evaluate the treatment results base on multicenter data, evaluate the prognostic factors that influence the radiological and clinical outcome, and also propose a treatment strategy for such type of fracture pattern.

## Materials and methods

From July 2011 to July 2016, pediatric patients with forearm fractures were retrospectively reviewed at seven high-volume geographically separated pediatric trauma centers. The inclusion criteria were (1) patients with olecranon fracture with an associated ipsilateral radial neck fracture, (2) availability of complete clinical and radiological data, and (3) had completed a minimum of 24 months follow-up. The exclusion criteria were (1) metabolic bone disease and (2) open fracture or fracture-dislocation, or concomitant fracture of the upper extremity.

The demographic data, fracture characteristics, type of treatment method, and postoperative data, including clinical and radiological outcomes and complications, were collected from seven centers. The type of reduction and surgical technique utilized was determined by reviewing operative reports. This study was approved by the ethical review committee of Tongji Medical College. All guardians of patients signed written informed consent, although the data were collected anonymized and centrally.

### Radiographic and clinical evaluation

Radiographic evaluation included an anteroposterior (AP) and a lateral view X-ray of the operated elbow. Each patient did not undergo computed tomography (CT) scan, but if the patients had undergone CT scans of the injured elbow for any reason, the three-dimensional reconstruction pictures had been given priority for classification. The fracture was classified based on anatomical and biomechanical considerations, including the obliquity of the fractures in the coronal and sagittal plane, degree of fracture separation, presence of comminution of the olecranon, and associated degree of displacement or angulation of the redial neck.

In every follow-up visit, patients were evaluated using MEPS and Flynn criteria [14, 15]. The clinical evaluation included the passive range of motion (ROM) of the elbow and the carrying angle. Any complications, including neurovascular complications, reduced ROM, any evidence of superficial, and deep infection were also recorded. Evidence of fracture union was evaluated by postoperative radiographs taken in each of the follow-up visits. Delayed union was considered if the fracture was not united in 3 months postoperatively.

### Statistical analysis

Statistical analysis was performed using STATA/SE software (version 12.0; STATA/SE, TX, USA). Fisher’s exact test was used to analyze the correlation between functional outcome and olecranon or radial neck fractures subtypes and the correlation between functional outcome and treatment choices. Significance was defined as *P* < 0.05.

## Results

**Fig. 1:**
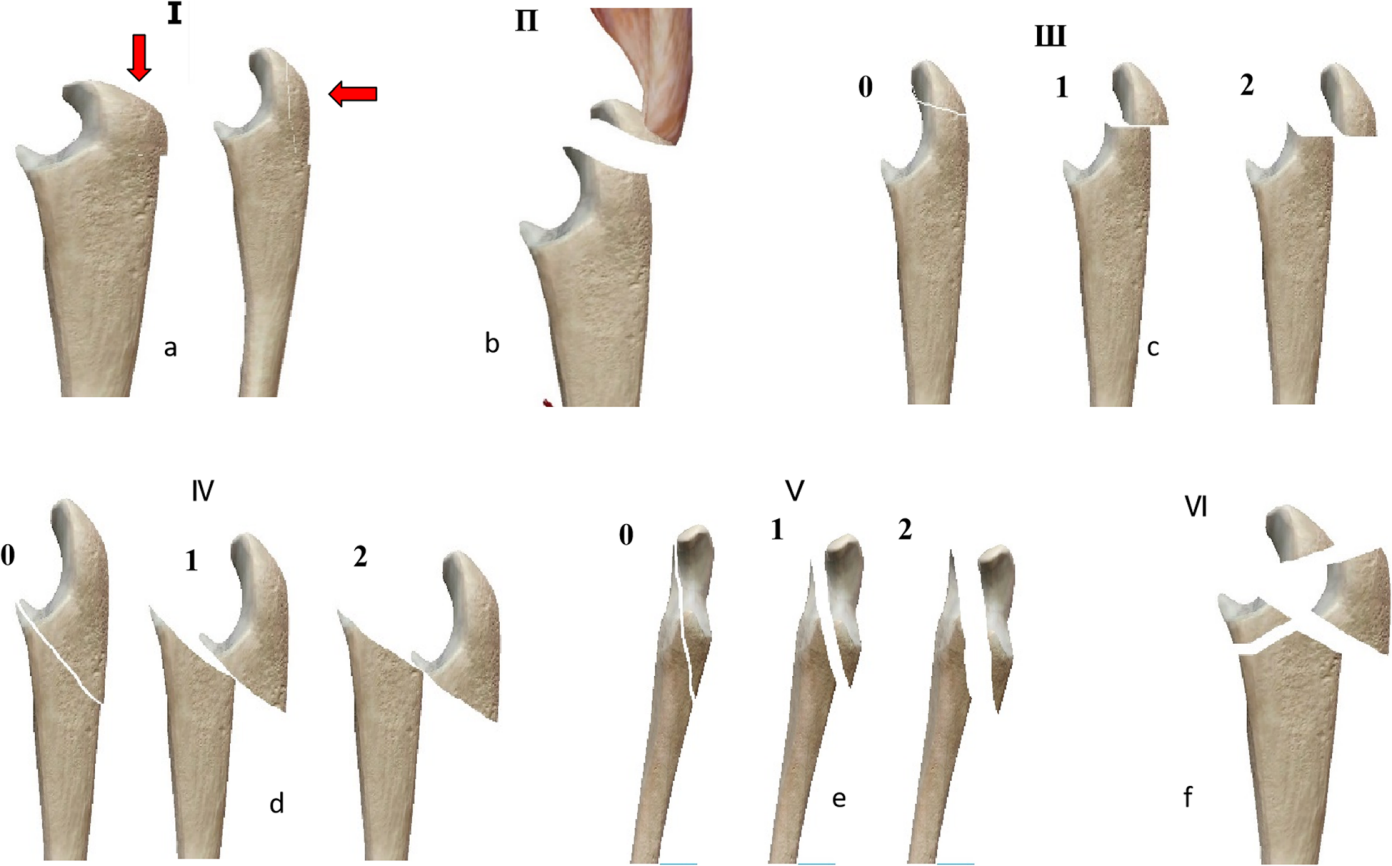
Characterization of olecranon fracture types. **a** Type I, longitudinal or transverse compression; **b** type II, olecranon avulsion fracture; **c** type III, transverse proximal to coronoid process; **d** type IV, oblique through coronoid process; **e** type V, coronal fracture; and **f** type VI, comminuted fractures (degree of displacement: 0, displacement less than one third ulnar width; 1, between one third and two thirds; 2, over two thirds of ulna)

**Fig. 2:**
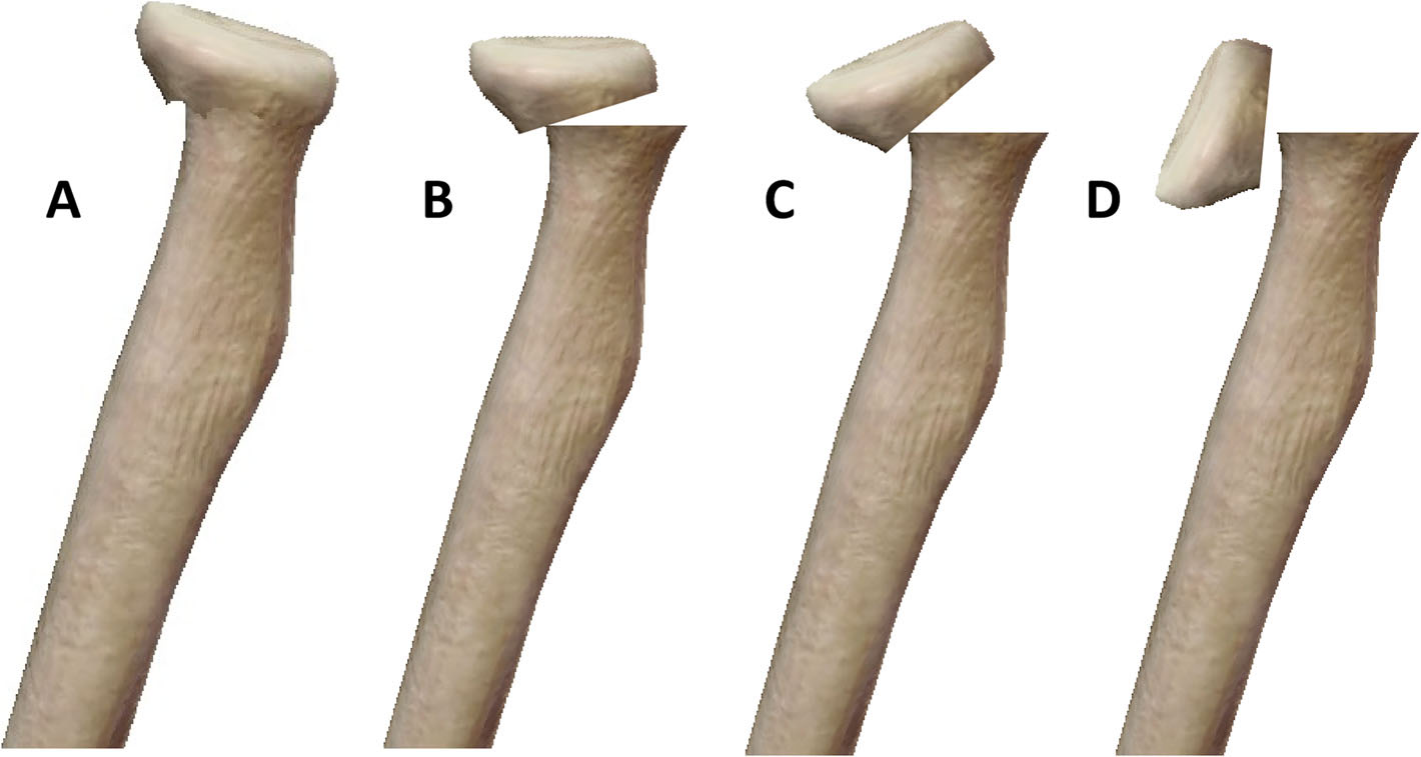
Characterization of radial neck fractures. **a** Longitudinal compression of radial neck without angulation or displacement, **b** fracture with angulation between 0 and 30° or displacement less than one third of radial neck width, **c** fracture with angulation between 30 and 60° or displacement between one third and two thirds of radial neck width, and **d** fracture with angulation more than 60° or displacement more than two thirds of radial neck width

**Fig. 3:**
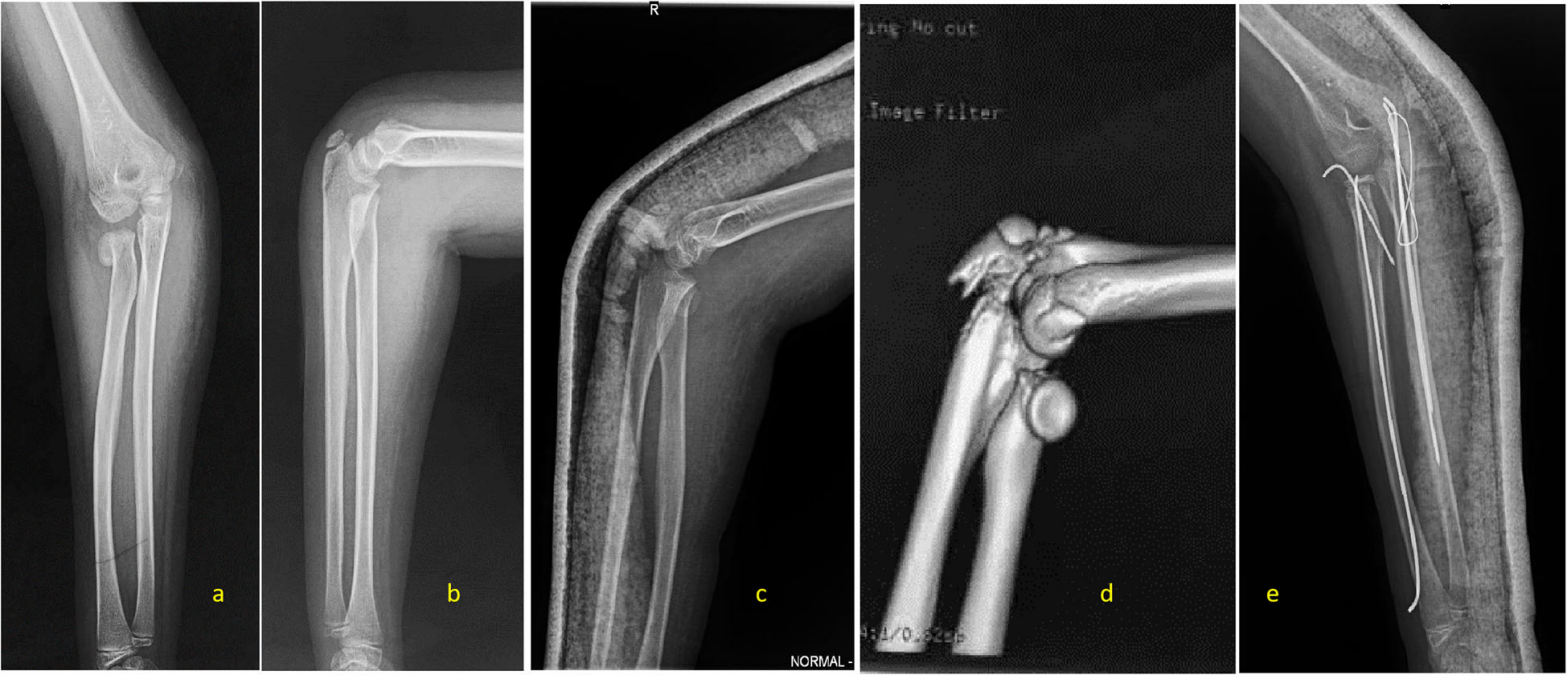
An 8-year-old girl with a type II injury in right elbow. **a**, **b** A-P and lateral view of X-ray film show transverse olecranon fracture proximal to the coronoid process without displacement, associated with radial neck fracture with angulation more than 60° or displacement more than two thirds of radial neck. **c** After 1 week plaster immobilization of the elbow, lateral view of X-ray film shows displacement of olecranon fracture, and a type III 2D fracture. **d** 3D reconstructed image of CT scan shows the precise fracture configuration. **e** After open reduction and internal fixation with K-wires for the olecranon and close reduction and internal fixation with a flexible nail and cross pinning for the radial neck

**Fig. 4:**
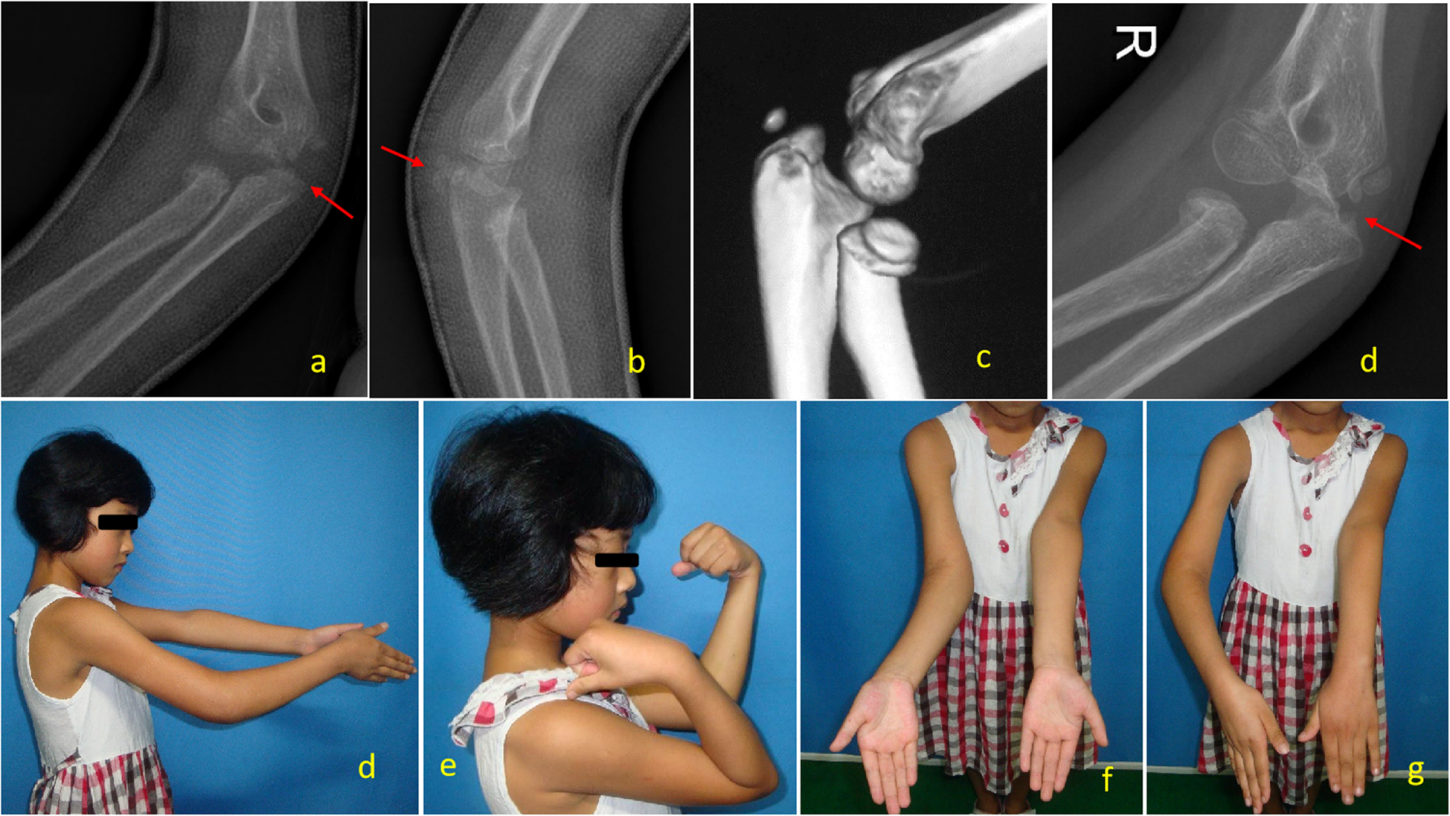
A 9-year-old girl with a type III injury in right elbow. **a**, **b** A-P and lateral view of X-ray film show olecranon avulsion fracture associated with a radial neck fracture angulated more than 30° and displacement between one third and two thirds of radial neck width. **c** 3D reconstructed image of CT scan demonstrate the complexity of the injury. **d** AP X-ray at 3 months, treated with 4 weeks of plaster immobilization only, demonstrating malunion of olecranon. **e**, **f** Twelve months after injury, the limitation of extension and **g**, **h** pronation of right elbow and forearm were observed

**Fig. 5:**
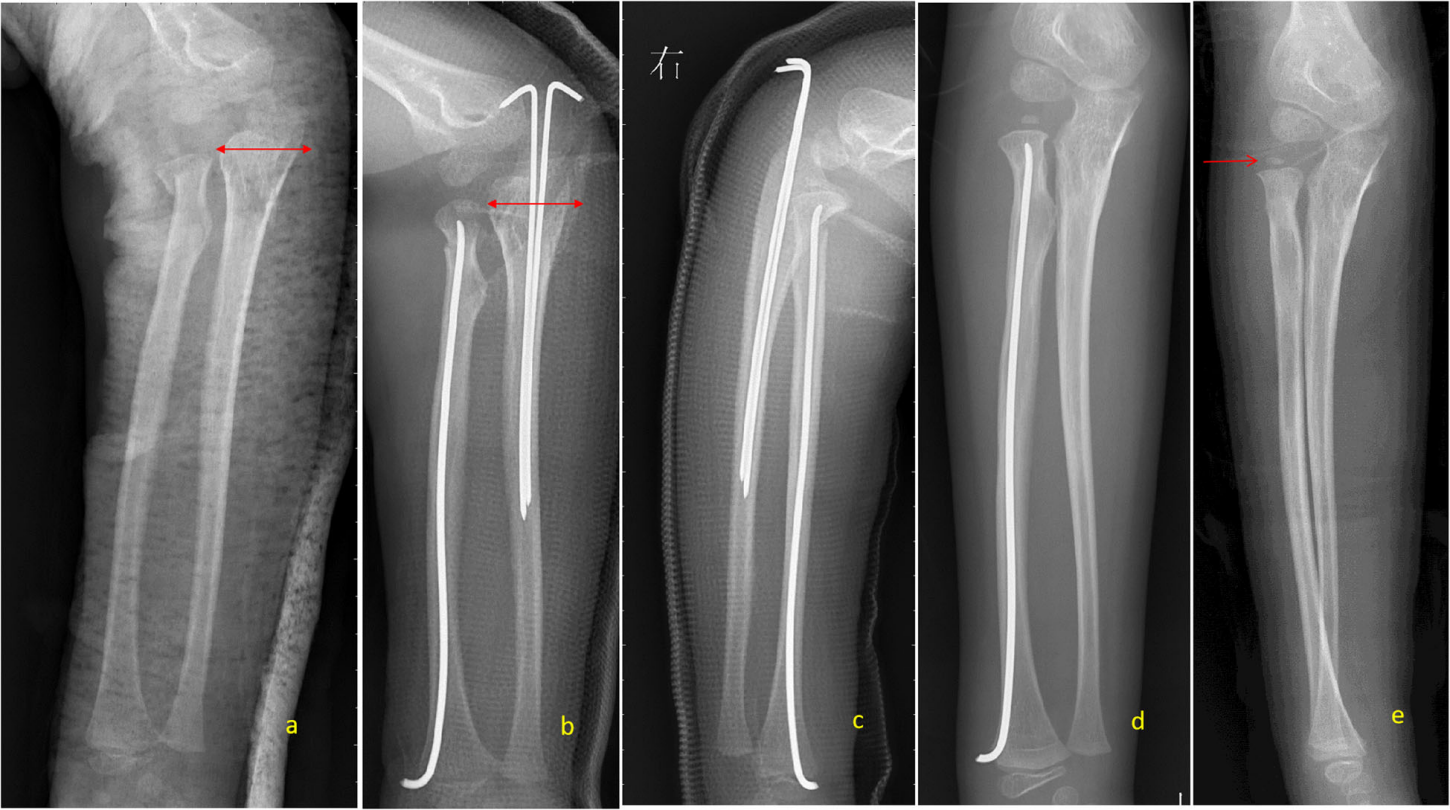
A 7-year-old boy classified type IV injury in right elbow. **a** A-P view of X-ray film show fracture in coronal plane of olecranon with displacement less than one third of ulna (arrow) associated with radial neck fracture with angulation less than 60°. **b**, **c** A-P and lateral view of X-ray film show this boy was treated with close reduction and internal fixation with K-wire for olecranon and close reduction and internal fixation with ESIN for radial neck, but the broad of olecranon in coronal plane was not corrected (arrow). **d** Three months postoperation, A-P view of X-ray film show the radial neck fracture healed but malunion of olecranon. **e** Six months postoperation, A-P view of X-ray film show radial head subluxation could be observed after the ESIN removed because of the angulated malunion of olecranon when pronate, only pronation of right forearm was limited

**Fig. 6:**
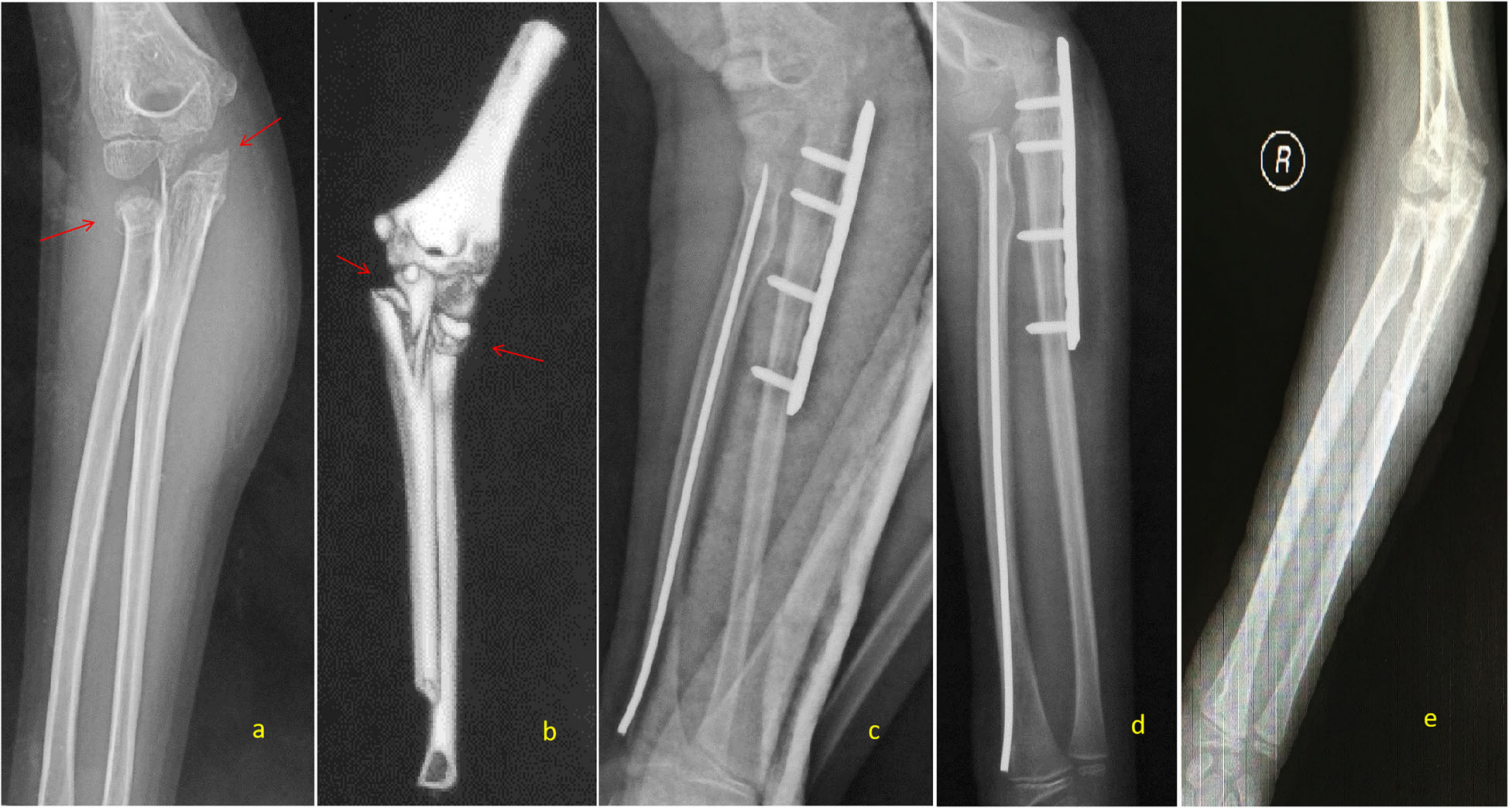
A 9-year-old boy classified type IV injury in right elbow. **a** A-P view of X-ray film show fracture in coronal plane of olecranon with displacement less than two thirds of ulna (arrow) associated with radial neck fracture with angulation less than 30°. **b** 3D reconstructed image of CT scan show the precise injury complex. **c** A-P view of X-ray film shows this boy was treated with open reduction and internal fixation with locking-plate for olecranon and close reduction and internal fixation with ESIN for radial neck; the broad of olecranon in coronal plane was corrected. **d** Three months postoperation, A-P view of X-ray film show the olecranon and radial neck fracture healed without malunion. **e** Six months postoperation, A-P view of X-ray film shows no radial head subluxation could be observed after the ESIN and plate removed; the flexion, extension, pronation, and supination were normal.

Out of 8292 forearm fractures, 137 patients were identified with ipsilateral olecranon and radial neck fractures. These patients included 83 (60.6%) males and 54 (39.4%) females with an average age of 7.5 years (1.5 to 14.8). The fracture occurred on the right side in 68 patients (49.6%) and the left side in 69 patients (50.4%). One hundred twenty-five patients with an average of 36 months (24–64 months) follow-up were available for the final evaluation. With the olecranon, six fracture subtypes were identified: (1) longitudinal or transverse compression (Fig. 1a), (2) olecranon avulsion (Fig. 1b), (3) transverse proximal to the coronoid process (Fig. 1c), (4) oblique through the coronoid process (Fig. 1d), (5) coronal (Fig. 1e), and (6) comminuted fractures (Fig. 1f). Longitudinal or transverse compression fractures, the classic greenstick fracture of pediatric olecranon fractures, had compression in the coronal and sagittal plane. The olecranon avulsion fracture involves disruption of the triceps insertion. The transverse fracture proximal to the coronoid process originates and ends proximal to the coronoid process and is associated with varying degrees of displacement (less than one third, between one third and two thirds, over two thirds of the ulnar width). Oblique fractures through the coronoid process may originate proximal or distal to the coronoid but will exit through the coronoid. It is also accompanied by varying degrees of displacement (less than one third, between one third and two thirds, over two thirds of the ulnar width). Coronal fractures involve a longitudinal disruption of the olecranon, with or without displacement (less than one third, between one third and two thirds, over two thirds of the coronal ulnar width). Comminuted fractures include fractures in any plane and had varying degrees of fragment displacement. Within the radial neck fractures, 4 subtypes were identified: (1) axial compression of the radial neck without angulation or displacement (Fig. 2a), (2) fracture with angulation between 0 and 30° or displacement less than one third of radial neck width (Fig. 2b), (3) fracture with angulation between 30 and 60° or displacement between one third and two thirds of radial neck width (Fig. 2c), and (4) fracture with angulation more than 60 ° or displacement more than two thirds of radial neck width (Fig. 2d). All the patients included in this study could be categorized by this expanded classification system. Most of the olecranon fractures were I to IV subtypes (130, 95% of all cases). Thus, olecranon fracture subtypes I to IV, with radial neck fractures subtypes A to D, were included in our statistical analysis. There was no statistically significant association between the olecranon and radial neck fracture subtypes (*p* = 0.054, Table 1). There was statistically significant in increasing average ages of olecranon fractures, with different fracture subtypes (*p* = 0.032, Table 2). From olecranon subtypes, I to IV, the average ages of patients increased gradually. In radial neck fractures subtypes, no statistically significant difference was found in the average ages (*p* = 0.091, Table 2). All fractures healed without infection, nonunion, or myositis ossificans between 8 and 12 weeks after the operation. No iatrogenic nerve injuries and residual vascular deficits were noted.

**Table 1:** Distribution of radius and ulna fracture types, *N* (%)

Ulna	Radius				
	A	B	C	D	Total
I	8 (22.2)	18 (50)	5 (13.9)	5 (13.9)	36
II	4 (9.5)	21 (50)	6 (14.3)	11 (26.2)	42
III	1 (4.5)	4 (18.2)	6 (27.3)	11 (50)	22
IV	5 (16.7)	12 (40)	6 (20)	7 (23.3)	30
Total	18	55	23	34	130

Fisher’s exact test, *P* = 0.054

**Table 2:** Age comparison between subtypes

		*N*	Age, mean (SD)	*P* value
Ulna	I	36	6.50 (2.45)	0.032
	II	42	7.31 (2.54)	
	III	22	8.27 (3.4)	
	IV	30	8.32 (2.99)	
Radius	A	18	7.88 (3.09)	0.091
	B	55	6.9 (2.79)	
	C	23	7.2 (2.95)	
	D	34	8.41 (2.59)	

ANOVA test

Fractures with a greater angulation or displacement of the olecranon (II to VI) with an associated radial neck (B to D) were associated with differences in the choice of surgery options. These fractures were associated with more surgical intervention for either the olecranon or radial neck, or both (*p* < 0.05, Table 3). Surgical treatment was the intervention of choice, particularly with type II to IV olecranon fractures or type B to D radial neck fractures. More than 90% of these fractures had surgical intervention. The incidence of each fracture subtype and the surgery options is listed in Table 3.

**Table 3:** Surgical intervention, single, or both bone

		*N* (%)			*P* value
		None	Single	Both	
Ulna	I	2 (5.5)	19 (52.8)	15 (41.7)	0.002
	II	4 (9.5)	6 (14.3)	32 (76.2)	
	III	1 (4.5)	4 (18.2)	17 (77.3)	
	IV	0 (0)	7 (23.3)	23 (76.7)	
Radius	A	4 (22.2)	6 (33.3)	8 (44.5)	0.02
	B	2 (3.6)	19 (34.6)	34 (61.8)	
	C	1^a^ (4.4)	5 (21.7)	17 (73.9)	
	D	0 (0)	6 (17.6)	28 (82.4)	

Single, ulna or radius; both, ulna and radius

Fisher’s exact test

^a^Choice made by patient’s parents

There was a significant difference in radius subtypes to correlate with a different outcome in the final Flynn functional (*p* = 0.015, Table 4). From the A to D radius subtypes, the final Flynn functional outcome of patients got worse gradually. Closed reduction of the radius had superior Flynn functional and MEPS (*p* < 0.001, Table 5) scores compared to those treated with open reduction. There was no same significant difference in olecranon between the subtypes (Tables 6 and 7). Only seven patients were classified as V to VI olecranon fracture subtypes according to this classification system. The treatment outcomes in these seven patients were excellent or good according to the Flynn and the MEPS criteria, except for one type V olecranon fracture. This particular patient’s coronal plane fracture was incompletely corrected, which resulted in a malunion.

**Table 4:** Distribution of radius subtypes according to criteria of Flynn and MEPS

Radius	Flynn functional, *N* (%)				MEPS, *N* (%)			
Satisfaction outcome	A	B	C	D	A	B	C	D
Excellent (0–5°/> 90)	15 (93.8)	35 (71.5)	12 (54.5)	21 (67.8)	15 (93.8)	43 (87.8)	15 (68.2)	23 (74.2)
Good (6–11°/75–89)	0 (0)	13 (26.5)	4 (18.2)	5 (16.1)	1 (6.2)	6 (12.2)	6 (27.3)	5 (16.1)
Fair (11–15°/60–74)	1 (6.2)	1 (2)	5 (22.8)	4 (12.9)	0 (0)	0 (0)	1 (4.5)	2 (6.5)
Poor (> 15°/< 60)	0 (0)	0 (0)	1 (4.5)	1 (3.2)	0 (0)	0 (0)	0 (0)	1 (3.2)
*P* value	0.015				0.186			

Fisher’s exact test

**Table 5:** Distribution of radius treatment options according to criteria of Flynn and MEPS

Radius	Flynn functional, *N* (%)			MEPS, *N* (%)		
Satisfaction outcome	Leave alone	Open	Close	Leave alone	Open	Close
Excellent (0–5°/>90)	16 (80)	6 (26.2)	61 (81.3)	18 (94.7)	8 (34.8)	70 (92.1)
Good (6–11°/75–89)	2 (10)	11 (47.8)	9 (12)	1 (5.3)	13 (56.6)	4 (5.3)
Fair (11–15°/60–74)	1 (5)	5 (21.7)	5 (6.7)	0 (0)	1 (4.3)	2 (2.6)
Poor (> 15°/< 60)	1 (5)	1 (4.3)	0 (0)	0 (0)	1 (4.3)	0 (0)
*P* value	< 0.001			< 0.001		

Fisher’s exact test

**Table 6:** Distribution of ulna subtypes according to criteria of Flynn and MEPS

Ulna	Flynn functional, *N* (%)				MEPS, *N* (%)			
Satisfaction outcome	I	II	III	IV	I	II	III	IV
Excellent (0–5°/> 90)	23 (69.7)	27 (69.2)	13 (68.4)	20 (74)	29 (88)	30 (76.9)	15 (78.9)	22 (81.5)
Good (6–11°/75–89)	8 (24.2)	8 (20.5)	4 (21)	2 (7.4)	3 (9)	8 (20.5)	3 (15.8)	4 (14.8)
Fair (11–15°/60–74)	1 (3)	3 (7.7)	2 (10.5)	5 (18.5)	1 (3)	0 (0)	1 (5.3)	1 (3.7)
Poor (> 15°/< 60)	1 (3)	1 (2.6)	0 (0)	0 (0)	0 (0)	1 (2.6)	0 (0)	0 (0)
*P* value	0.482				0.731			

Fisher’s exact test

**Table 7:** Distribution of ulna treatment options according to criteria of Flynn and MEPS

Ulna	Flynn functional, *N* (%)			MEPS, *N* (%)		
Satisfaction outcome	Leave alone	Open	Close	Leave alone	Open	Close
Excellent (0–5°/> 90)	17 (68)	11 (84.6)	55 (68.8)	20 (80)	11 (84.6)	65 (81.3)
Good (6–11°/75–89)	5 (20)	0 (0)	17 (21.3)	4 (16)	2 (15.4)	12 (15)
Fair (11–15°/60–74)	2 (8)	2 (15.4)	7 (8.7)	1 (4)	0 (0)	2 (2.5)
Poor (> 15°/< 60)	1 (4)	0 (0)	1 (1.2)	0 (0)	0 (0)	1 (1.2)
*P* value	0.403			0.984		

Fisher’s exact test

There was a variety of surgical fixation options utilized to address the olecranon or radial neck fractures. These implants included K-wires, screws, absorbable rods, elastic stable intramedullary nailing (ESIN), and locking plates. The MEPS and Flynn scores did not demonstrate that any one of these fixation options were superior.

## Discussion

To our knowledge, this is the first study to characterize this type of injury. Though there are other descriptions of Monteggia equivalents injuries [16–19], ipsilateral olecranon fractures with associated radial neck fractures were not included. Some previous studies have reported ipsilateral olecranon fractures with an associated radial neck fracture. However, most of these are from a single-center, with a small sample size, and combine these injuries with the classic Monteggia fractures [4, 5, 20–22]. With the existing literature and our series, we suggest that these ipsilateral olecranon fractures associated with proximal radius injuries should not be included in the discussion of radial head dislocations [23] and instead be separately described and classified. In the setting of olecranon fractures, the radial head usually dislocates only from the capitellum, while the proximal radio-ulnar joint is intact. This is reflected in our series. The former description reflects the absence of proximal radio-ulnar diastasis but rather a disruption of the radiocapitellar articular. The latter description indicates that both the proximal radio-ulnar joint and radiocapitellar joints are disrupted in a true Monteggia fracture-dislocation. If the distinction between these two terms could be made, it would enable clinicians to distinguish these types of injuries from classic Monteggia fracture-dislocations [24].

A classification with a total of 24 subtypes and distinguished these types of fracture from Monteggia injuries of radial head dislocations could be demonstrated according to the result in this study. But this anatomical and biomechanical classification was too complex to be used for treatment guidance or expectations for clinical outcomes. This multiple-center study aims to simplify the classification system for ipsilateral olecranon fractures with associated radial neck fractures with an assessment of the fracture characteristics, treatment, outcome, and complications. The new classification system demonstrates the pattern of treatment selection and clinical outcomes, thus supporting its use as a simplified classification system. Based on outcomes of 125 patients (average, 36 months; range, 24–64 months), we could propose the following treatment advice for these fractures of ipsilateral olecranon with an associated radial neck fracture. The classification with a total of 24 anatomical and biomechanical subtypes could be simplified to five subtypes: type I, ulna olecranon or radius angulation less than 30° or displacement less than one-third width; type II, ulna olecranon or radius angulation equal to or more than 30° or displacement equal to or more than one-third width; type III, ulna olecranon avulsion fracture; type IV, ulna olecranon coronal fracture; and type V, ulna olecranon comminuted fractures. Only type I fracture could be reserved for non-operative treatment. Type II to V could be advised for operative treatment (Table 8).

**Table 8:** Simplified classification of ipsilateral radial neck associated with olecranon fractures according to the treatment strategies

Type	Ipsilateral radial neck associated with olecranon fractures	
**I**	Ulna or radius angulation < 30° or displacement < 1/3	Not operate
**II**	Ulna or radius angulation ≥ 30° or displacement ≥ 1/3	Operate
**III**	Ulna olecranon avulsion fracture	Operate
**IV**	Ulna olecranon coronal fracture	Operate
**V**	Ulna olecranon comminuted fractures	Operate

If ulna olecranon or radius fractures were classified over type I, these fractures tend to be unstable. Even only ulna olecranon or radius was diagnosed as type II fracture, but another one without displacement, the elbow lacks its inherent stability, and displacement is likely to occur. In the series of this study, an 8-year-old girl was with a type II injury, transverse nondisplaced olecranon fracture above the ulna coronoid process, associated with radial neck fracture with angulation more than 60°. After 1 week of plaster immobilization of the elbow, follow-up radiographs demonstrated both ulna olecranon and radius displacement. This girl was treated with open reduction and internal fixation with K-wire for olecranon and close reduction and internal fixation with ESIN and K-wire for the radial neck (Fig. 3). That is also the reason why the treatment strategies formulated in this study only recommend type I could be treated without surgery.

Type III ulna olecranon avulsion fractures should be treated surgically. The triceps insertion can cause displacement of the olecranon and thus close follow-up is warranted even with non-operative treatment. In our series, a 9-year-old girl with a type III injury (Fig. 4), olecranon avulsion with a radial neck fracture of over 30° of angulation, underwent non-operative treatment as per parents’ preference. She had 4 weeks of cast immobilization, and at 3 months, radiographs demonstrate malunion of the olecranon avulsion. With the malunion, impingement of the olecranon over the humeral trochlea was there. Twelve months after the injury, a limitation of elbow extension and pronation of the forearm was observed.

Type IV ulna olecranon fracture in the coronal plane should be corrected with the effective surgical technique because this type of olecranon fracture has tendency to get malunion [13]. Stable and effective internal fixation is essential to corrected olecranon fracture in the coronal plane, which could avoid radial head subluxation because of olecranon malunion and ensures high-quality functional recovery [25]. Two cases included in this study showed different outcomes depending on whether the broad of the olecranon in the coronal plane was corrected or not. One was a 7-year-old boy classified as type IV injury in the right elbow. X-ray film showed a fracture in the coronal plane of olecranon with displacement less than one third of ulna associated with radial neck fracture angulation less than 60°. This boy was treated with close reduction and internal fixation with K-wires for olecranon and close reduction and internal fixation with ESIN for radial neck, but the olecranon in the coronal plane was not corrected. Three months after the operation, X-ray film showed the radial neck union but malunion of the olecranon. Six months postoperation following the removal of ESIN, X-ray film showed radial head subluxation because angulated malunion of olecranon during pronation and pronation of right forearm was limited (Fig. 5). The other was a 9-year-old boy classified type IV injury on the right elbow. X-ray film showed the fracture in the coronal plane of olecranon with displacement less than two thirds of ulna associated with radial neck fracture with angulation less than 30° and 3D reconstructed CT scan images the precise injury complex. This boy was treated with open reduction and internal fixation with locking-plate for olecranon and close reduction and internal fixation with ESIN for radial neck, the broad of the olecranon in coronal plane was corrected. Three months postoperation, the olecranon and radial neck fracture united without malunion. Six months postoperation, X-ray film showed no radial head subluxation following the implant removal. The flexion, extension, pronation and supination movement were normal. Stable and effective internal fixation is essential to correct olecranon fracture in the coronal plane, which could avoid radial head subluxation because of olecranon malunion and ensures high-quality functional recovery [25] (Fig. 6).

**Fig. 7:**
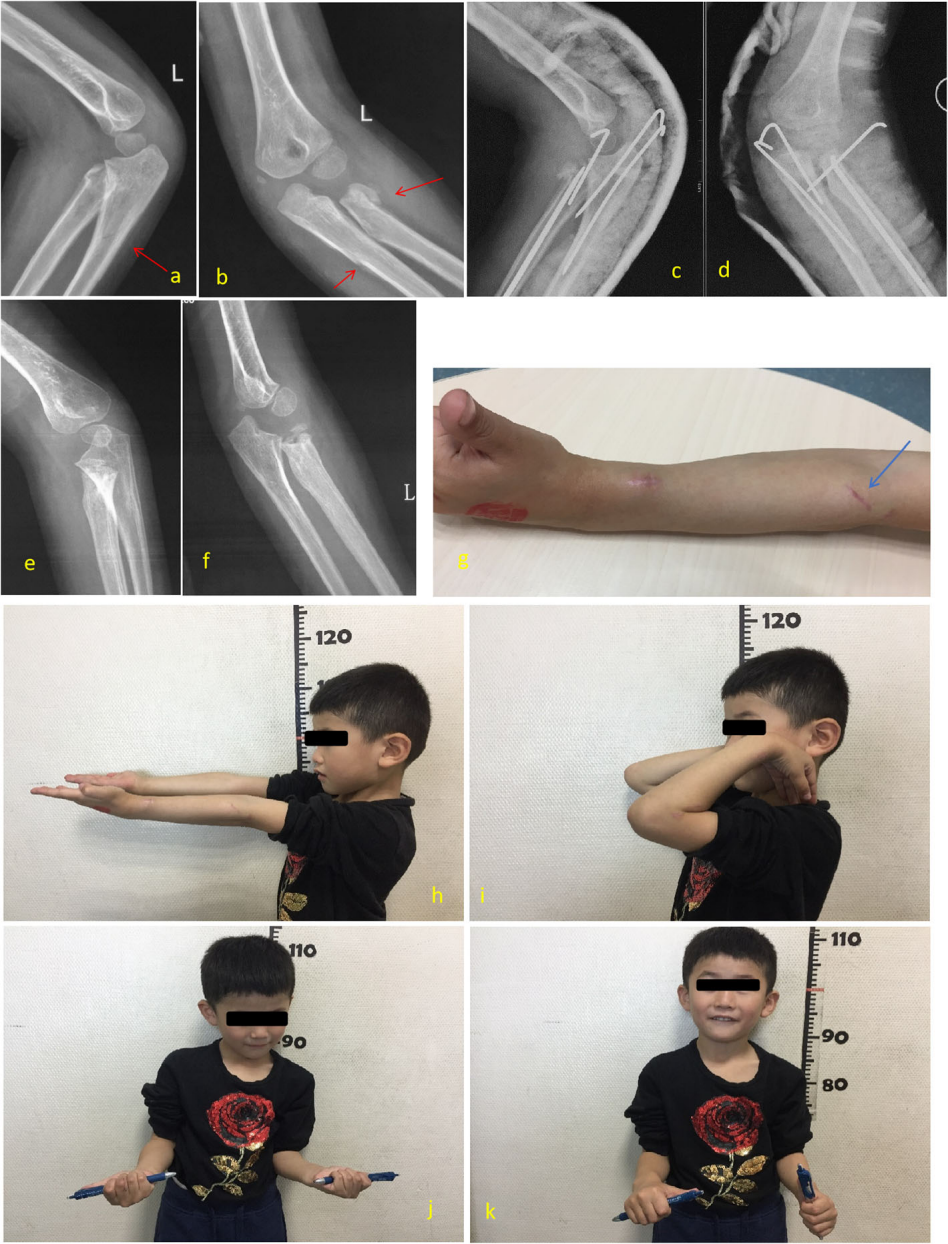
A 5-year-old boy classified type II injury in right elbow. **a**, **b** Lateral and A-P view of X-ray film show oblique through ulna coronoid process without displacement of ulna (arrow) associated with radial neck fracture with angulation more than 60°. **c**, **d** Lateral and A-P view of X-ray film show this boy was treated with close reduction and internal fixation with K-wire for olecranon, but open reduction and internal fixation with K-wires and ESIN for radial neck. **e**, **f** Six months postoperation, A-P and lateral view of X-ray film show the olecranon and radial neck fracture healed with ischemic change of radial head after K-wires and ESIN removed 3 months postoperation. **g** Mini incision for open reduction of radial neck fracture (arrow). **h**–**k** Twelve months postoperation, the flexion, extension, and supination were normal but pronation was limitated in left forearm

If olecranon injuries were type III, type IV, or type V, with type I radial neck injuries, only ulna olecranon fracture needs operative treatment. For treating the associated radial neck fractures, we recommend closed reduction as the first-line intervention. Although radius subtypes tended to have worse outcomes in final Flynn functional outcomes according to the increasing severity of displacement or angulation (Table 4), close reduction also correlated with better Flynn functional and MEPS outcomes (Table 5). Open reduction, which has the risk of compromising the blood supply to the radial head, should be reserved for severely displaced fractures or failed close reduction. One case of a 5-year-old boy, our series demonstrates open reduction may lead to poor functional outcomes such as limitation of pronation [10, 26–29] (Fig. 7). Open surgery should be avoided whenever possible and that closed methods should be attempted first, and a less-than-anatomic reduction may be accepted rather than opening.

The authors acknowledge that there are several limitations to the study. Despite the multiple-center series, we did have certain subtypes with very limited numbers, which precluded statistical analysis. The clinical results of this study are limited to mid-term follow-up, but many early complications are noted within the 6-month period. This is a retrospective study, and the interobserver and intraobserver reliability of this simplified classifications system still needs to be tested. As a retrospective multiple-center study with surgeon-chosen treatment, there were confounding factors between injury type and treatment type before this classification was proposed. Even within surgical classes, there were many different treatments given. Therefore, it is not powered to assess the best types of treatment for these many types of classes. However, we believe the findings are significant as it is the largest series of ipsilateral olecranon with associated radial neck fractures from multiple different centers. Both of these could be well addressed in the future with a prospective multiple-center study.

## Conclusion

Standardization of treatment and discussion of ipsilateral olecranon fractures with associated radial neck fractures is a challenge because this type of injury is uncommon in previous reports. Based on the first standardized description of this type of injury and noting its unique properties, this study assessed the fracture characteristics, treatment, outcome, and complications. This retrospective multiple-center study with a patient pool of more than 8000 forearm fractures not only simplified the classification, but also provides an opportunity to predict mid-term treatment outcomes and choices for treatment selection. Orthopedic surgeons could be more easy to avoid complications and poor outcomes by knowing this type of fracture. In their clinical practice, appropriate treatment choices for this type of injury could be selected by evaluating the treatment strategies proposed in this study.

## Data Availability

The datasets used and/or analyzed during the current study are available from the corresponding author on reasonable request.

## Abbreviations

MEPS: Mayo elbow performance score
AP: Anteroposterior
CT: Computed tomography
ROM: Range of motion
ESIN: Elastic stable intramedullary nailing
